# Myalgia: A Clinical Study

**Published:** 1918-02-02

**Authors:** 


### SOME SUBJECTIVE EVIDENCES OF DISEASE.

### Myalgia: a Clinical Study.

Among the minor difficulties of medical practice a prominent place has to be found for cases in which there are subjective symptoms unaccompanied by any objective explanation. The patient complains of, say, pain, but on examination the physician fails to find any abnormal condition capable of causing the pain. The symptom is affirmed, but there is no physical fact capable of confirming or explaining it. Such a position is a frequent one, and it is apt to be specially puzzling to the young practitioner. During his hospital experience—whether as a student or a resident—he has had to deal mainly with cases in which physical signs of disease are conspicuous, and while he gives due attention to the patient's history and sensations he is accustomed to rely for his diagnosis mainly on the conditions which he discovers for himself on examining the patient. Hence it is not altogether surprising when he steps into a position where cases of all sorts and degrees confront him that he should be somewhat nonplussed to find complaints for which the most careful investigation fails to discover any adequate physical cause. Such cases give him some very unhappy moments. If he happens to hold a modest and sensitive view of his own capacity he is inclined to fear that the failure to discover a cause is due to his own inexperience or incompetence. With a more confident view of his powers his tendency is to believe the patient guilty of exaggeration or of pretence. In either event he is at his wits' end for a diagnosis, and lacks, therefore, a reasonable guide to treatment. To a large extent such cases are new to him, for they have been but a minor feature in his previous hospital experience. He will, however, make a great mistake if he pushes them to one side as having little interest or reality, for they will form a large proportion of his future work, and on his ability in dealing with them will depend in large measure his reputation and success.

### Perseverance in Diagnosis.

The study is a difficult one, and this for several reasons. In a certain proportion of these cases there is exaggeration and pretence. In others the patient's loquacity and irrelevance strain the patience even of the most long-suffering of physicians. In not a few time alone will afford the interpretation. And at the best there will remain a certain number to which no clear and confident diagnostic label can possibly be attached. Yet, in spite of these hindrances, the physician who aims at efficiency must resolutely persevere. He has to learn to distinguish, say, a pain which is the first signal of some serious and deep-seated mischief from one which, though annoying to the patient, has no anxious meaning. And among these latter he has to recognise varieties which differ from one another in kind and demand different forms of treatment. What the patient calls "rheumatism" may be the warning note of an intrathoracic aneurism or of an impending tabes dorsalis. On the other hand, a pain described in tragic tones as "pleurisy" may be nothing more than an intercostal neuralgia or an irritable condition of the thoracic muscles. This careful study of symptoms will have its reward. If less dramatic and less confident than the study of physical signs it is certainly not less important, and it affords an opportunity for the cultivation of keen observation and subtlety of judgment and for the development of the tact which is necessary to the gentle persuasion of a talkative and irrelevant patient. The art of diagnosis is largely a matter of interpretation, and clearly one of its opportunities is afforded by the sensations of the patient.

### Captain Chavasse's Investigations.

A very useful and apt illustration of the principles here stated may be found in a recent study of "myalgia," by Captain B. Chavasse, R.A.M.C., in the *British Medical Journal* for December 22, 1917. Taking a considerable number of cases sent back from the Front, Captain Chavasse applied to them a more careful examination than had been possible prior to the preliminary diagnosis, and he thus endeavoured to arrange in appropriate groups cases which had in common one feature—namely, the existence of pain without any obvious physical explanation. Such an enterprise has obviously great importance relative to men serving with the Army. First, it may succeed in separating the malingerer from the real sufferer, and so may contribute to the maintenance of man-power. In the second place, a differential diagnosis will indicate the treatment required and thus may help to restore the efficiency of the sufferers and to make them competent for return to duty. And, yet again, an examination conducted with the object of exact interpretation will be likely to indicate that, though unsuitable for certain forms of service, the soldier may yet undertake other duties. In all these respects the Army is served, and this is an important aim for the Army medical officer. At the same time the sufferer gains the best chance of appropriate treatment and full relief, which, after all, is the main business of medicine. If together with such an end the Army is helped so much the better. But even the Army doctor must remember that his first duty as a practitioner of medicine is to the individual patient. To cure the man is the demand of medicine; it is the law, not medicine, which makes him a soldier and bids him, when cured, return to the ranks. In civil practice there is no possibility of any confusion of motives, for the patient earnestly desires to get relief, and indeed this is the sole purpose which brings him to the doctor's consulting-room.

Now we may take a rapid view of Captain Chavasse's inquiry and of the clinical and numerical results which it displays. He summarises 200 cases, each one of which came back to the

**SOME SUBJECTIVE EVIDENCES OF DISEASE**—(*continued*).

field-ambulance carrying the diagnostic label "myalgia." In nineteen of these he found that the pain of which the patient complained was accompanied by pyrexia, which, of course, means that the diagnosis of myalgia was inadequate to the facts. Muscular pains are common experiences when the bodily temperature is above the normal; they are then only symptomatic of some general condition, and the diagnosis must be sought in an exhaustive examination. In several of the cases quoted the raised temperature was present only when the record was taken in the evening, the morning reading being normal or subnormal. Clearly such patients if examined at a morning sick parade would not be recognised as other than the victims of muscular pains, and hence would be grouped as cases of myalgia. The hint is one which the practitioner should not overlook. There is something more to be said about the possible association of muscular pains with febrile disturbance. Such pains may continue after the fever has quite disappeared, and only the history of the case will give the correct interpretation. This history may have the precise quality of a temperature chart, but often it has to be inferred from the patient's story of shivering, headache, sweating, etc., on some date shortly before he comes under observation. Presumably the prejudicial effects of the high temperatures, or of the toxins from which these arise, continue for a time and are exhibited in the form of muscular pains. These post-pyrexial pains can be distinguished from those of simple or true myalgia by the fact that they are aggravated when the patient is warm in bed and is lying motionless. The pains of true myalgia, on the other hand, are absent during complete rest. They appear with muscular movements, but gradually cease as the patient by persistence gets rid of the stiffness which rest induces. From this distinction arises a guide to treatment, for while myalgia calls for Turkish baths, massage, liniments, and so on, the post-pyretic pains contra-indicate these methods: they call rather for rest and for the other tonic measures which promote convalescence.

Other groups of Captain Chavasse's patients taken out of the 200 "myalgics" are arthritis and sciatica. The former may be readily overlooked if the joint changes are slight, but an examination for creaking, limitation of movement, and muscular wasting should never be omitted. Sciatica, clearly, ought not to be mistaken, as the site of the pain, tenderness over the nerve trunk, and pain on testing for Kernig's sign, present a decided diagnostic picture.

Taking now the more positive side, Captain Chavasse endeavours to determine what are the actual facts on which a diagnosis of myalgia may be surely based. In his own experience at the Front it is of interest to note that he found anything like gross malingering decidedly uncommon. Some exaggeration was not infrequent, but this could be said with equal emphasis of civil practice. At the Front it is obvious the conditions are readily calculated to produce gloomy views in men of a lugubrious disposition, and these will be reflected in the men's statements of their own sensations. The British soldier has a traditional reputation for cheerfulness under difficulties, and the present war has in no way impaired this reputation. But we must allow for exceptions, as the philosophical temper and the resolute heart are not the possession even of every man who wears the King's uniform. Given, then, the complaint of pain, how shall the case be justly cast into the myalgic category? First, it is to be noted, the pain may exist in one of many situations. In Captain Chavasse's cases the lumbar region, the sacral region, and the buttocks were the most common site, but in many instances the pain was referred to the limbs; in the latter an exact definition of the affected muscles was not attempted. A second point is that the pain of myalgia is usually symmetrical, as, for example, existing in the lumbo-sacral region and extending for a varying distance downwards into each thigh. Yet, here also, there are exceptions, and few would deny that pleurodynia is often, or even usually, unilateral. Much stress is placed on the character of the pain and the influence of various circumstances on it. So long as the patient is warm in bed and is quite still pain is absent. But a quick movement excites an acute stabbing sensation; rest develops a sense of stiffness in the affected muscles, and this gradually wears off under the influence of moderate activity. The condition is aggravated by cold and damp weather, and again by prolonged exertion. Its onset is often sudden, and recurrences frequently affecting one and the same region of the body are not uncommon. An important point is that massage aggravates the pain, and yet, as a method of treatment, is decidedly beneficial.

With this clinical sketch of myalgia Captain Chavasse presents a second, which he holds is that, not of myalgia, but of neuralgia. Here the pain is described as aching, gnawing, or throbbing; so far from being relieved by rest in bed and immobility it is aggravated by these conditions; the patient has difficulty in getting sleep, and there is usually considerable cutaneous anæsthesia. Most writers would probably say that if a pain is neuralgic it is usually unilateral and that certain painful points may be found over the affected area. Possibly the distinction here is of academic rather than of practical concern, but the exercise is a stimulus to clear thinking and is decidedly to be encouraged. In any event a careful clinical study is to be welcomed. The ambition is a much more difficult one than that cultivated by the laboratory worker, and it by no means always receives an adequate measure of recognition. Yet whate'er machines and tests emerge it must always remain true in medicine that the patient is the centre of the inquiry and that his actual condition must be determined by the personal intervention of the physician. Captain Chavasse's careful work is a study on clinical lines, and we have read it with much interest.

